# Comparative analysis of myeloperoxidase deficiency in humans and mice: Conserved and species-specific effects on neutrophil biology

**DOI:** 10.1016/j.redox.2026.104386

**Published:** 2026-09-09

**Authors:** Maysam Ahdab, Henning Guthoff, Nathalie Nix, Sonja Derman, Anna Greta Barbe, Harshal Nemade, Elvina S. Philip, Kezia Singgih, Kathrin Nellessen, Alina Hüttermann, Adnana Paunel-Görgülü, Chris Diekmann, Friedrich F. Hoyer, Patrick Schelemei, Jan-Wilm Lackmann, Stefan Müller, Wibke Johannis, Thomas Streichert, Stephan Baldus, Holger Winkels, Philipp von Stein, Martin Mollenhauer, Alexander Hof

**Affiliations:** a Heart Center, Department of Cardiology, Faculty of Medicine and University Hospital Cologne, University of Cologne, Cologne, Germany; b Center for Molecular Medicine Cologne, University of Cologne, Cologne, Germany; c Faculty of Medicine and University Hospital Cologne, Polyclinic for Operative Dentistry and Periodontology, University of Cologne, Cologne, Germany; d Heart Center, Department of Cardiac Surgery, Faculty of Medicine and University Hospital Cologne, University of Cologne, Cologne, Germany; e Institute of Clinical Chemistry, University of Cologne, Cologne, Germany

**Keywords:** Myeloperoxidase, MPO deficiency, Neutrophil biology, Innate immune system

## Abstract

**Background:**

Myeloperoxidase (MPO), a heme-containing enzyme released by activated polymorphonuclear neutrophils (PMNs) represents a key mediator of inflammation-driven cardiovascular disease, positioning MPO as a promising therapeutic target. However, substantial interspecies differences between murine and human neutrophil biology challenge the translation of preclinical findings from rodent studies into patients.

**Methods:**

11,608 patients were screened for MPO deficiency using the ADVIA 2120i system. 282 individuals exhibited a reduced MPO activity. 56 patients were eligible, consented to be enrolled and were compared to 20 control patients. MPO levels and activity were measured by enzyme-linked immunosorbent assay (ELISA), immunoblotting, a tetramethylbenzidin (TMB)-based peroxidase and nitric oxide (NO)-consumption assay. For cross-species analysis, shotgun proteomics, Gene Ontology (GO) and REACTOME pathway enrichment analyses were conducted on bone marrow derived PMNs from *Mpo*^*−/−*^ mice and human MPO-deficient PMNs.

**Results:**

Patients with persistent (MPO^low^, *n* = 12) and transient (MPO^rec^, *n* = 44) MPO deficiency were identified. MPO^low^ individuals showed significantly decreased neutrophil MPO concentrations, enzymatic activity, and plasma MPO levels. Proteomic profiling revealed substantial overlap in altered biological processes across both species. Species-specific enrichments included humoral immunity, complement activation, and coagulation in humans, phagocytosis, chemotaxis, and transcriptional regulation in mice. These differences have been reported alongside distinct signaling patterns, with predominance of Mitogen-activated protein (MAP)-kinase and cytokine activity in humans, and Ras homolog family (Rho)-GTPase pathways in mice.

**Conclusion:**

MPO deficiency leads to broadly conserved alterations in neutrophil immune and stress responses across humans and mice. However, similar functional outcomes may involve different signaling pathways in each species contributing to divergent phenotypes in disease models. These findings underscore species-specific mechanisms in MPO-related neutrophil biology and highlight critical considerations for translating MPO-targeted therapies from animal models to human disease.

## Highlights

• 11,608 individuals were screened for persistent MPO deficiency, enabling direct human-mouse comparisons.
• Proteomic profiling of PMN reveals conserved alterations in neutrophil immune and stress responses across humans and mice.
• MPO-regulated pathways included humoral immunity and complement in humans versus phagocytosis and chemotaxis in mice.
• Species-specific differences have been reported alongside MAPK and cytokine activity in humans and Rho GTPase pathways in mice.

## Graphical abstract

In a hospital-based cohort, 11,608 individuals were screened for neutrophil MPO activity, identifying patients with persistent MPO-deficiency (MPO^low^, *n* = 12) and transient MPO deficiency (MPO^rec^, *n* = 44). Persistent MPO deficiency was associated with decreased MPO protein expression, enzyme activity and plasma levels. Proteomic profiling of human MPO^low^ and murine *Mpo*^*−/−*^ PMNs revealed conserved alterations in neutrophil stress responses, innate immune activation, inflammatory signaling and cell death across both species. Species-specific enrichments included humoral immunity, complement activation, and coagulation in humans, phagocytosis, chemotaxis, and transcriptional regulation in mice. TLR/MAPK/JNK signaling was reduced in MPO^low^, whereas Rho GTPase-dependent pathways were altered in murine *Mpo*^*−/−*^ PMNs. Together, these findings suggest that similar functional outcomes associated with MPO deficiency may involve distinct signaling pathways in each species, potentially contributing to divergent phenotypes in disease models.

**Figure:**
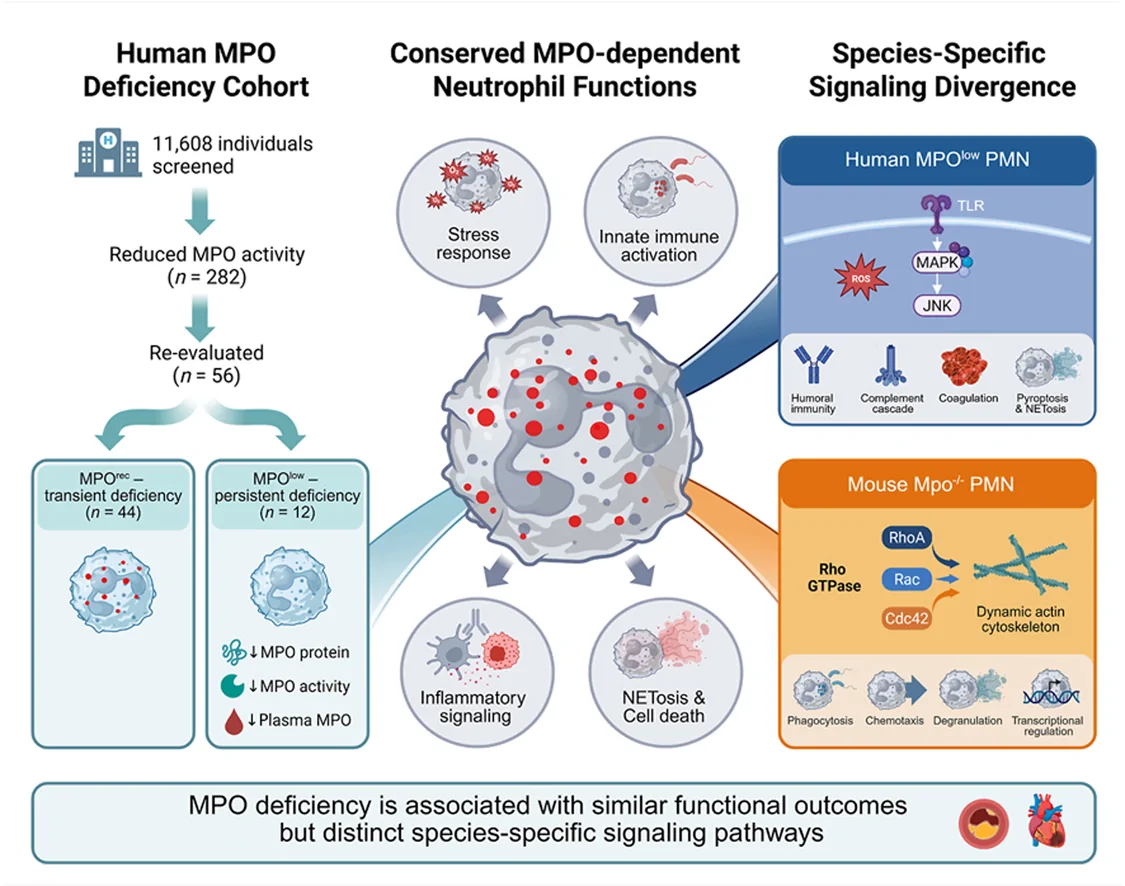

## 1 Introduction

Cardiovascular diseases (CVDs) remain the leading cause of mortality worldwide accounting for approximately one-third of all global deaths [1]. Vascular inflammation is a key driver of disease progression contributing not only to atherosclerosis, but also to various CVD phenotypes such as heart failure with reduced or preserved ejection fraction (HFrEF, HFpEF) and pulmonary hypertension (PH). Under these conditions, immune cell activation and oxidative stress promote endothelial dysfunction, maladaptive cardiac remodeling and progressive ventricular impairment [[2], [3], [4]]. Given the substantial individual and socioeconomic burden of CVDs, there is an urgent need for innovative therapeutic strategies to prevent or attenuate disease progression.

Polymorphonuclear neutrophils (PMNs) have emerged as central mediators of vascular inflammation. Myeloperoxidase (MPO), a heme-containing enzyme abundantly expressed in PMNs, has gained particular attention due to its dual role in host defense and immune regulation. MPO contributes to microbial killing by generating reactive oxidants such as hypochlorous acid (HOCl) and modulates neutrophil effector functions, including oxidative burst, cytokine signaling, and the formation of neutrophil extracellular traps (NETs). Furthermore, MPO-derived oxidants can also promote vascular injury and tissue damage [5,6].

Elevated plasma MPO levels in humans have been consistently associated with worse clinical outcomes across multiple CVD phenotypes [7,8]. In HFrEF and HFpEF, MPO levels correlate with New York Heart Association (NYHA) functional class, N-terminal pro–B-type natriuretic peptide (NT-proBNP) levels, and impaired left ventricular function [9,10]. In PH, increased MPO levels are linked to pulmonary vascular remodeling and adverse prognosis [11]. Genetic polymorphisms that reduce MPO activity are associated with decreased cardiovascular risk and mortality underscoring MPO's potential as both a biomarker and a therapeutic target [12].

Despite these promising clinical associations, translation of MPO-targeted therapies from murine models to patients has proven challenging. Preclinical studies using MPO-deficient mice (*Mpo*^−/−^) have produced conflicting results. While reduced MPO levels in humans appears protective against cardiovascular morbidity [13,14], murine studies with *Mpo*^−/−^ mice have paradoxically reported increased atherosclerotic burden under hyperlipidemic conditions [15]. Conversely, mice with MPO-inhibition or genetic depletion exhibit improved endothelial and cardiac function and attenuated post-infarction remodeling [16,17], yet clinical trials using MPO inhibitors in heart failure patients did not fully replicate these protective effects [18,19]. Such inconsistencies raise important questions about the translatability of murine findings to the human disease. Human neutrophils have increased MPO expression compared to mouse neutrophils and differ in granule composition, receptor expression, and effector responses [20,21]. Consequently, the molecular and functional consequences of MPO deficiency observed in *Mpo*^−/−^ mice may not accurately reflect human physiology, limiting their predictive value for therapeutic development.

In this study, we aim to explore how MPO deficiency affects molecular and functional properties of neutrophils in humans and mice, and to identify potential species-specific differences. By comparing these aspects of neutrophil biology, we hope to gain a better understanding of MPO's immunomodulatory role in cardiovascular inflammation. These observations may help to place preclinical findings into context and inform the interpretation of MPO-targeted approaches, ultimately supporting their safe and effective translation into clinical practice.

## 2 Methods

### 2.1 Human studies

A total of 11,608 consecutive patients undergoing assessment of the white blood cell count at the University Hospital of Cologne were screened for reduced MPO activity using the ADVIA 2120i Hematology Analyzer. MPO deficiency was defined as a mean peroxidase index (MPXI) ≤ −18, based on established literature and manufacturer specifications. Accordingly, 282 subjects exhibiting a MPXI below the threshold of −18 were considered eligible for this study, from whom 76 individuals agreed to participate in a follow-up examination two years after index screening. Patients older than 18 years and free of overt infections (as evidenced clinically by C-reactive protein (CRP) < 5 ng/mL and interleukin-6 (IL-6) < 10 ng/L) were eligible to be enrolled. Based on the above exclusion criteria, 56 subjects were finally included. MPO-competent controls (*n* = 20) were recruited from the University Hospital Cologne Outpatient Clinic and were not subject to longitudinal follow-up. Controls were matched for age, sex, arterial hypertension, smoking status, low- and high-density lipoprotein (LDL/HDL), body mass index (BMI), and glycated hemoglobin (HbA1c). All enrolled and screened subjects gave written informed consent. The study was registered in the German Clinical Trials Register (DRKS00019108) and approved by the Ethics Committee of the Cologne University (Az19-1580).

### 2.2 Human blood sampling and processing

Peripheral blood was collected into EDTA-coated S-Monovettes for plasma isolation, PMN purification and PBMC isolation, and into heparin-coated S-Monovettes for routine clinical chemistry. Plasma was separated by centrifugation at 1500 rcf for 10 min at room temperature (RT) and stored at −80 °C.

PMNs were isolated using density gradient centrifugation. Whole blood was mixed 1:1 with 3% Dextran in 0.9% NaCl, sedimented for 35 min at RT. The leukocyte-rich supernatant was layered onto an equal volume of Histopaque-1077 Samples were centrifuged at 600 rcf for 30 min without brake. PMNs and erythrocytes formed a pellet, which was resuspended in 5 mL sterile distilled water to lyse erythrocytes. Samples were centrifuged (350 rcf, 10 min, 4 °C) after addition of 35 mL 0.9% NaCl. The cell pellet was either frozen at −80 °C or used fresh for downstream analyses. PBMCs were isolated using density gradient centrifugation. Whole blood was diluted 1:1 with phosphate-buffered saline (PBS) and gently layered over an equal volume of Lymphoprep**™** (Stemcell Technologies). After centrifugation at 900 rcf for 20 min at RT without brake, the PBMC layer (interphase) was carefully aspirated, washed with PBS, and centrifuged at 1500 rcf for 10 min at RT. The resulting pellet was resuspended in RPMI 1640 medium (Thermo Fisher Scientific) supplemented with GlutaMAX**™** (Thermo Fisher Scientific) and 10% fetal bovine serum (FBS) (Sigma-Aldrich). Cells were cryopreserved in freezing medium (15% dimethyl sulfoxide (DMSO) in FBS) at a 1:1 ratio in cryovials and stored at −80 °C.

### 2.3 Quantification of neutrophil MPO expression

Protein concentration of human and murine PMN samples was determined using the Pierce™ 660 nm Protein Assay (Thermo Fisher). Neutrophil MPO concentration was measured using the Human Myeloperoxidase ELISA Kit (R&D Systems, DY3174) and Mouse MPO ELISA Kit (Hycult Biotech, HK210).

Human samples were diluted 1:2000 in ELISA buffer (PBS + 0.05% Tween 20 + 0.1% BSA), and absorbance was measured at 450 nm (Multiskan FC, Thermo Fisher). For Western blotting, PMNs were lysed in radioimmunoprecipitation assay (RIPA) buffer. Equal amounts of protein were separated on 4–20% gradient sodium dodecyl sulfate (SDS)-polyacrylamide gels (Bio-Rad), transferred to nitrocellulose membranes and probed with rabbit anti-MPO (1:1,000, Sigma) and anti-GAPDH (1:1,000, Cell Signaling), followed by horseradish peroxidase (HRP)-conjugated goat anti-rabbit immunoglobulin G (IgG) (1:5,000, Vector Labs) and enhanced chemiluminescence (ECL) detection.

### 2.4 Quantification of neutrophil MKK4 expression

Neutrophil MAP2K4 (MKK4) concentration was measured with the Human MKK4 ELISA Kit (AAA Biotech, AAA123515). Absorbance was measured at 450 nm (Multiskan FC, Thermo Fisher).

### 2.5 MPO activity quantification

Neutrophil MPO activity was assessed using a TMB-based assay. PMNs (1 × 10^5^ cells) were lysed in 930 μL NaH_2_PO_4_ buffer (12.48 g/L, pH 5.5), 60 μL TMB solution (48 mg TMB in 10 mL N,N-Dimethylformamide) was added, and baseline absorbance was recorded. The oxidative reaction was initiated with 10 μL of 10 mM H_2_O_2_. Afterwards, the absorbance at 655 nm was measured every 30 s for 3 min using a NanoDrop™ 2000c.

MPO-dependent NO consumption was measured by determining the NO-redox potential. 1 × 10^6^ PMNs were suspended in 1 mL PBS, and the baseline NO-redox potential was recorded. The suspension was supplemented with 10 μL of 10 mM propylamine NONOate (PAPA-NONOate, NO donor), after which the NO potential was measured (Pmax). MPO was activated with 5 μL 10 mM H_2_O_2_, and NO consumption was monitored in real time until a stable redox potential (Pmin) was reached. The percentage decrease, ΔP = (Pmax − Pmin)/Pmax, was used to quantify NO consumption and derive neutrophil MPO activity. All measurements were performed using the APOLLO 4000 Free Radical Analyzer.

### 2.6 MPO plasma protein quantification

Plasma MPO concentration was measured with the Human Myeloperoxidase ELISA Kit (R&D Systems, DY3174) and Mouse MPO ELISA Kit (Hycult Biotech, HK210). Plasma samples were diluted 1:20 in ELISA buffer (PBS + 0.05% Tween 20 + 0.1% BSA), and absorbance was measured at 450 nm (Multiskan FC, Thermo Fisher).

### 2.7 MPO genomic DNA amplification and sanger sequencing

Genomic DNA was isolated from PBMCs using the PureLink™ Genomic DNA Mini Kit (Thermo Fisher Scientific) according to the manufacturer's instructions. The MPO gene was amplified from approximately 200 ng genomic DNA using a HiFi PCR polymerase mix (Takara) in 50 μL reactions. Three overlapping genomic PCR fragments covering exons 1-6, 8-10, and 11-12 were generated using the primer sets and conditions summarized in **Sup.** Table S1. PCR products were separated by agarose gel electrophoresis, excised, and purified using the NucleoSpin Gel and PCR Clean-up kit (Macherey-Nagel). Purified amplicons were subjected to Sanger sequencing by Eurofins Genomics using internal sequencing primers (**Sup.** Table S1).

Sequencing chromatograms were analyzed using BioEdit and Benchling and aligned to the human MPO genomic sequence (Ch.17, GRCh38.p14, Ensembl: ENSG00000005381). Coding exons were inspected for sequence variants, with particular attention to previously reported MPO mutations. Exon 7 could not be successfully amplified and was therefore not included in the sequence analysis.

### 2.8 Murine PMN isolation from bone marrow

Murine studies were performed within a genetic MPO-deficient mouse model first described by Brennan 2001 [22]. *Mpo*^*−/−*^ (B6.129X1-Mpotm1Lus/J, JAX stock #004265) mice were obtained from and are available at The Jackson Laboratory (USA) and further bred in our animal facility. Littermates were used as controls. PMNs were isolated from murine bone marrow using a discontinuous Percoll density gradient. Femurs and tibias were harvested from 8 to 12-week-old male mice following euthanasia by cervical dislocation. After removal of residual tissue, bones were flushed with Hanks’ balanced salt solution (HBSS) supplemented with 3% FBS (HBSS prep), and the bone marrow suspension was filtered through a 70 μm cell strainer. Cells were centrifuged at 400 rcf for 5 min at 15 °C. Erythrocytes were lysed by adding 6 mL of 0.2% NaCl for 45 s at RT and isotonicity was restored by addition of 14 mL 1.2% NaCl. After centrifugation at 400 rcf, 5 min, 15 °C, the cell pellet was resuspended in 5 mL HBSS and layered on top of 5 mL 62% Percoll (diluted from isotonic Percoll stock solution with HBSS). Samples were centrifuged at 1000 rcf for 30 min at 15 °C without brake. PMNs were collected from the pellet, resuspended in 1 mL HBSS with 3% FBS, washed at 400 rcf, 5 min, 15 °C, and finally resuspended in PBS. The cells were either frozen at −80 °C or used fresh for downstream analyses. All animal studies were approved by the local authorities (Ministry for Environment, Agriculture, Conservation and Consumer Protection of the State of North Rhine-Westphalia: State Agency for Nature, Environment and Consumer Protection (LANUV), NRW, Germany with the authorization number: 81-02.04.2018.A216) and by the University of Cologne Animal Care and Use Committees.

### 2.9 Flow cytometric assessment of PMN isolation purity

Purity of the isolated PMN fractions was assessed by flow cytometry using a BD LSRFortessa flow cytometer. For mouse samples, PMNs were isolated from bone marrow collected from femurs and tibiae and then stained with DAPI as a live/dead marker, BV711-conjugated anti-CD45 (1:700 dilution), APC-conjugated anti-Ly6G (1:700), and APC-Cy7 conjugated anti-CD11b (1:700). Isolated mouse PMNs were identified as live CD45^+^ CD11b + Ly6G + cells.

For human samples, PMNs were isolated from blood and stained according to a neutrophil staining strategy based on Schofield et al. in Cytometry Part A. The isolated human PMN fraction was stained with FVS780 live/dead dye (1:640), R718 conjugated anti-CD45 (1:80), PE/Fire640-conjugated anti-CD66b (1:80), and BV786 conjugated anti-CD15 (1:160), and human PMNs were identified as viable CD45^+^CD15^+^CD66b + leukocytes.

### 2.10 Shotgun proteomics and bioinformatics

Proteomic analysis of isolated human and murine PMNs was performed in collaboration with the CECAD/ZMMK Proteomics Facility (University of Cologne, Germany). PMNs were homogenized in 8 M urea lysis buffer supplemented with protease inhibitors using the Precellys 24 Touch homogenizer (Bertin Technologies). Lysates were centrifuged at 14,000 rcf for 10 min and the supernatant was further disrupted with the Bioruptor 300 (Diagenode). Protein concentration was determined using the Pierce™ 660 nm Protein Assay (Thermo Fisher) after serial dilution.

Equal protein amounts (50 μg in 50 μL) were reduced with 2.5 μL of 100 mM dithiothreitol (DTT), alkylated with 5.25 μL of 550 mM chloroacetamide (CAA), and diluted with 50 mM TEAB buffer. Samples underwent overnight digestion with trypsin (enzyme:substrate ratio 1:75). Digestion was stopped by acidification with formic acid. Peptides were purified using SDB-RP StageTips conditioned with methanol and buffer solutions. Tips were dried with compressed air and stored at 4 °C.

Samples were analyzed on an Exploris 480 coupled either to an UltiMate 3000 or Vanquish neo nLC system (all Thermo Scientific). Samples were loaded onto an Acclaim PepMap C18 trap cartridge (#160434, Thermo Scientific) with a 15 μl flow of eluent A (0.1% formic acid) before reverse-flushed onto a 75 μm inner diameter, 30 cm pulled-tip column packed in-house with C18 material (Poroshell, Agilent). Peptides were separated on a 90 min gradient with linear increasing buffer B (80% acetonitrile, 0.1% formic acid) ramping from 2% B to 35% B in 73 min, to 55% B in 7 min, followed by column washing at 95% B and equilibration to starting conditions. The mass spectrometer was run in data-independent acquisition covering the range of 400 to 1000 *m*/*z* in 60 x 10 *m*/*z* windows at 15k resolution or 30 x 20 *m*/*z* windows at 30k resolution. AGC targets were at 100% forMS1 and 1000% for MS2 scans with maximum injection time set to 22 mn and 54 ms, respectively.

Data analysis was conducted in DIA-NN 1.8.1 [23] using match-between-runs function. The canonical Uniprot Human (downloaded 26/08/2020) or murine (downloaded 15/01/2024) reference proteome was used for analysis, fragment data simulated directly in DIA-NN using mass-range settings fitting to acquisition parameters. Samples were searched in double-pass mode and output filtered for at least six fragments per precursor before performing LFQ quantification in the DIA-NN R-package. Only protein identified with at least two (human PMN data set) or one (murine myeloid data set) unique peptides were used for downstream analysis in Perseus 1.6.15 [24]. Here, data was filtered for valid values in replicate groups, followed by imputation and FDR-controlled T-tests. Identified proteins were subjected to GO term enrichment and Reactome pathway analysis using the PANTHER platform.

### 2.11 Statistical analysis

Gaussian mixture modeling (GMM) was applied to neutrophil MPO expression data, modeling the observed data as a mixture of two Gaussian distributions to classify patients as persistently (MPO^low^) or transiently MPO-deficient (MPO^rec^). Categorical variables were expressed as percentages and compared using Fisher's exact test. Continuous variables were presented as mean ± standard error of the mean (SEM). Normality was tested with D'Agostino–Pearson test. Normally distributed variables were analyzed using Welch's ANOVA, whereas non-normally distributed data were assessed using the Kruskal–Wallis test. Correlations were evaluated using Spearman's rank correlation coefficient (r). A *p*-value < 0.05 was considered statistically significant. For multiple comparisons, the false discovery rate (FDR) was used to control for type I error accumulation. Data were analyzed using GraphPad Prism Version 10.

## 3 Results

### 3.1 Subclassification of MPO deficiency in human subjects

**Fig. 1:**
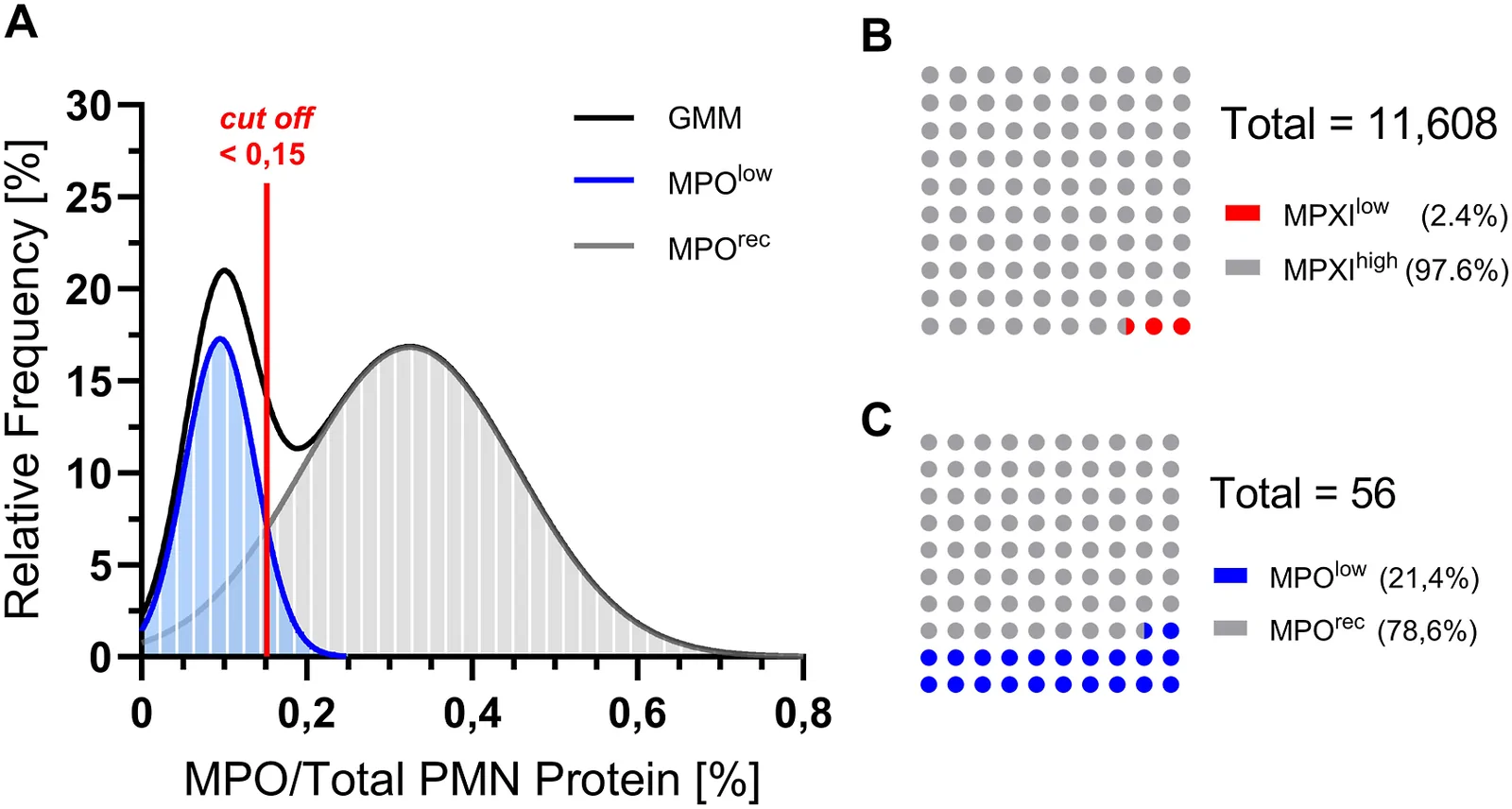
**Identification of persistent and transient MPO deficiency.** MPO protein expression was assessed in PMNs from 56 patients with reduced MPXI scores. Neutrophil MPO content (% of total PMN protein) showed a bimodal distribution, suggesting the presence of two distinct subpopulations. To characterize these populations, the data were modeled using a Gaussian mixture model (GMM) consisting of two Gaussian distributions, designated MPO^low^ and MPO^rec^. A cut-off value of ≤ 0,15% for the neutrophil MPO concentration was derived by intercept determination, which was utilised to subcategorise the patient cohort (**A**). Out of 11,608 patients, 282 individuals showed a reduced MPXI score (MPXI^low^) at the first examination (**B**). After a follow-up period of two years, 56 patients were reassessed, of which 12 patients showed persistent MPO deficiency (MPO^low^) and 44 patients showed physiological MPO protein expression (MPO^rec^), indicating transient MPO deficiency in the past (**C**). Point prevalence was estimated at 1:200 for persistent and 1:50 for transient MPO deficiency, respectively.

To systematically investigate human MPO deficiency, including its persistent and transient forms, a cohort of 56 patients was stratified based on neutrophil MPO content (% of total PMN protein). Measured values showed a bimodal distribution, indicating the presence of two underlying subpopulations. Accordingly, the data were modeled using a Gaussian mixture model (GMM), representing a probability density composed of two Gaussian distributions (**Sup.** Fig. S1A). A cut-off of ≤0.15% MPO concentration, derived from the intersection point of the fitted distributions, was used to stratify the cohort into MPO^low^ and MPO^rec^. (Fig. 1A). MPO-competent controls, recruited from the University Hospital Cologne Outpatient Clinic, showed a similar distribution of neutrophil MPO content (% of total PMN protein) compared to the MPO^rec^ group (**Sup.** Fig. S1B).

Among 11,608 screened patients, 282 individuals exhibited reduced MPXI scores (MPXI^low^) at the initial examination (Fig. 1B). After a two-year follow-up, 56 of these patients were reassessed: 12 maintained persistently low MPO expression (MPO^low^), while 44 showed normalized levels (MPO^rec^), indicating a transient deficiency in the past (Fig. 1C). The estimated point prevalence of persistent MPO deficiency was approximately 1:200, and 1:50 for transient deficiency. A detailed summary of the general causes of primary and secondary MPO deficiency and potential symptoms in affected patients is provided in **Sup.** Table S2.

### 3.2 Quantitative characterization of MPO deficiency

**Fig. 2:**
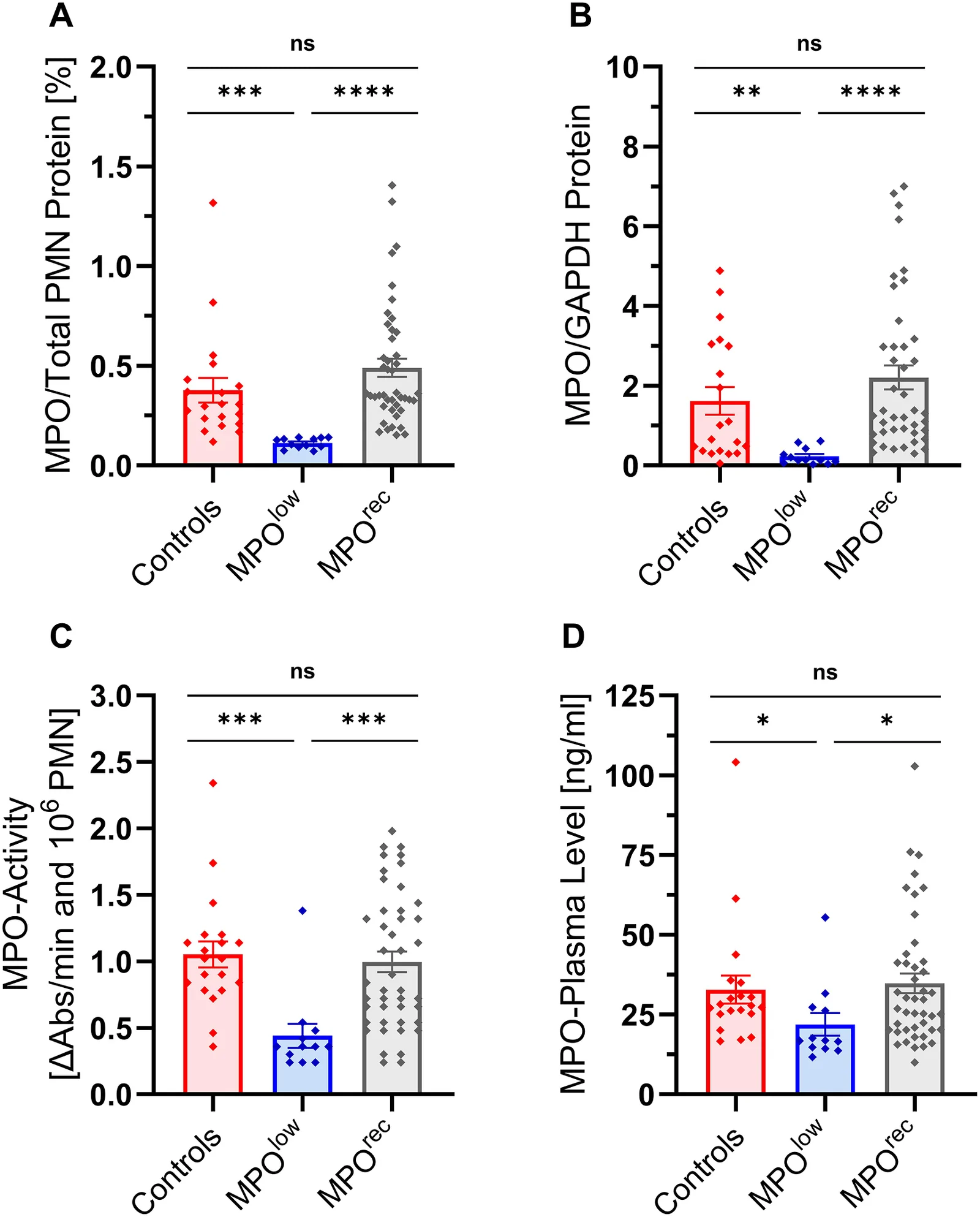
**MPO protein expression, activity, and plasma levels in MPO^rec^, MPO^low^ and control patients.** Photometric analysis by ELISA (**A**) and densitometric analysis by immunoblotting (**B**) were conducted on isolated PMN to determine MPO protein expression. Neutrophil peroxidase activity (**C**) and MPO plasma levels (**D**) were quantified. Results are shown for MPOrec (n = 44), MPOlow (n = 12) and control patients (*n* = 20). ****p < 0,0001; ***p < 0,001; **p < 0,01; *p < 0,05.

Neutrophil MPO protein expression, enzymatic activity, and MPO plasma levels were assessed in MPO^low^ (*n* = 12), MPO^rec^ (n = 44), and controls (*n* = 20). MPO^low^ PMNs exhibited markedly reduced MPO concentrations (0.11% ± 0.01 of total PMN protein) compared to MPO^rec^ (0.49% ± 0.05 of total PMN protein) and controls (0.38% ± 0.06 of total PMN protein, *p* < 0.01) (Fig. 2A). MPO^rec^ and control PMNs displayed comparable MPO levels (*p* = 0.40).

Immunoblot analysis confirmed these findings: relative MPO expression was significantly reduced in MPO^low^ PMNs (0.23 ± 0.06 rel. expression) compared to controls (1.62 ± 0.35 rel. expression, *p* < 0.01), while levels were similar between MPO^rec^ cells (2.21 ± 0.30 rel. expression) and controls (*p* = 0.44) (Fig. 2B–S3A). In two MPO^low^ patients, neutrophil MPO expression was below the detection threshold of the MPO ELISA (<0.08% of total PMN protein), and MPO bands were completely absent in immunoblot analysis (**Sup**. Fig. S2**).** Neither individual exhibited a clinical history suggestive of severe immunodeficiency or recurrent opportunistic infections (**Sup**. Table S3**).** DNA sequencing of the MPO gene identified in one MPO^low^ patient the MPO variant c.1705C > T, p.Arg569Trp (R569W) in exon 10, a variant associated with hereditary MPO deficiency (**Sup**. Fig. S4).

MPO peroxidase activity assessed by a TMB assay (Fig. 2C–S3B) was significantly lower in MPO^low^ PMNs (0.44 ± 0.09 TMB absorption/min/10^6^ PMNs) compared to MPO^rec^ (1.00 ± 0.08 TMB absorption/min/10^6^ PMNs, *p* < 0.01) and controls (1.05 ± 0.10 TMB absorption/min/10^6^ PMNs, *p* < 0.01). The number of eosinophilic erythrocytes in the blood count was comparable across all study groups, thereby excluding differential eosinophil-derived peroxidase activity as a potential confounder of the TMB assay (**Sup**. Fig. S3G**)**. The NO consumption by activated MPO was attenuated in MPO^low^ PMNs and correlated with TMB assessed MPO activity (**Sup**. Fig. S3C and S3D). Similarly, MPO plasma levels were decreased in MPO^low^ patients (21.90 ± 3.53 ng/ml) relative to both MPO^rec^ (34.64 ± 3.07 ng/ml, *p* = 0.02) and controls (32.80 ± 4.37 ng/ml, *p* = 0.04) (Fig. 2D–S3E). These findings demonstrate MPO deficiency on the cellular level, systemically, and functionally in MPO^low^ individuals. Interestingly, MPO-activity/protein ratios and plasma levels of inflammatory markers including CRP and IL-6 were similar between MPO^low^, MPO^rec^ and control patients (**Sup**. Figs. S3F, S3H, S3I**).**

### 3.3 Comparative proteomic profiling: human vs. murine MPO deficiency

**Fig. 3:**
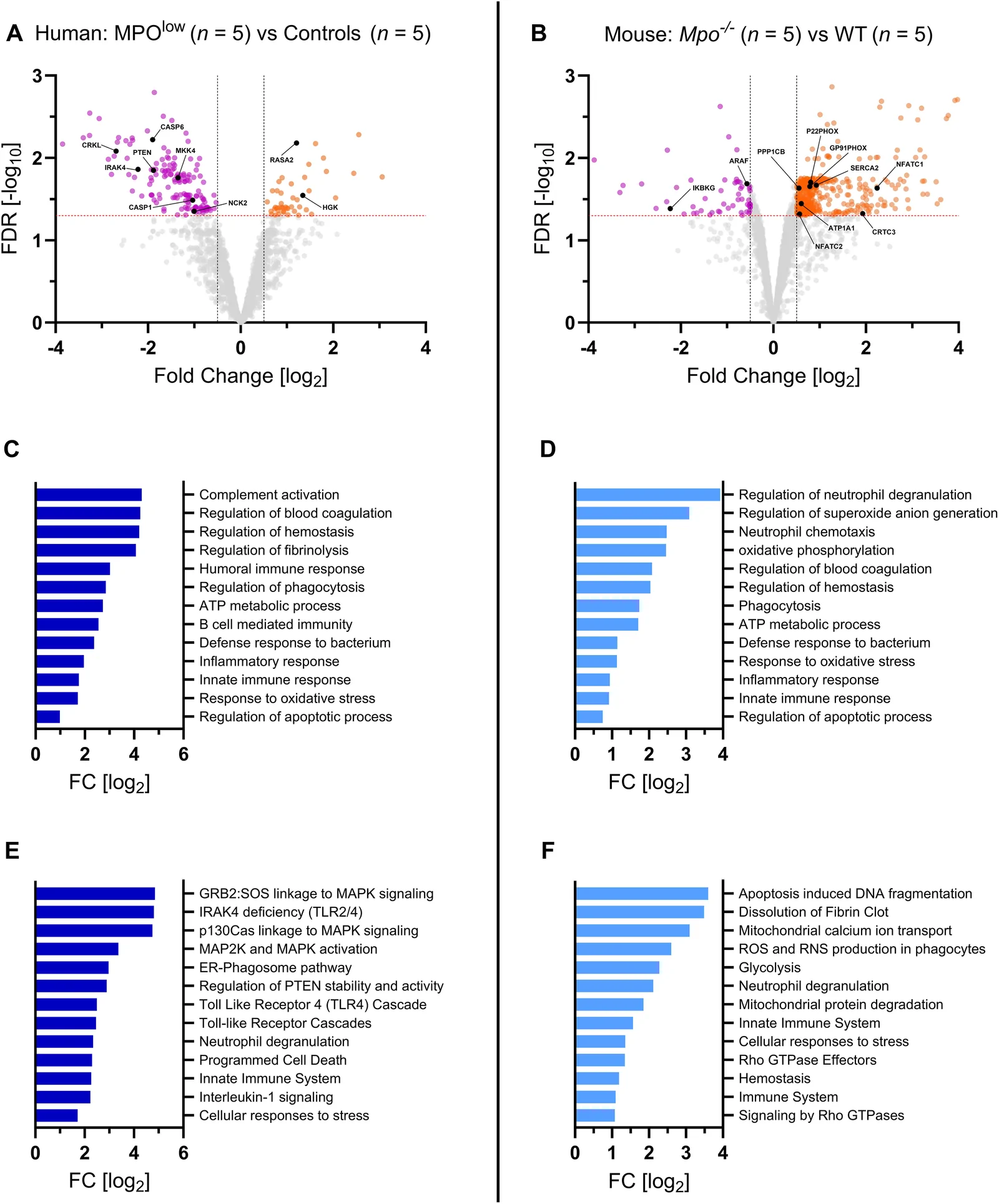
**Functional classification of differentially expressed proteins in human MPOlow and murine *Mpo*^*−/−*^ neutrophils.** Significantly regulated proteins in human and murine samples were identified based on Fold Change (FC) [log2] < −0.5; > 0.5 and False Discovery Rate (FDR) < 0.5. The results are displayed as volcano plots. In total, 204 proteins were significantly differentially regulated in human MPO^low^ PMNs (**A**) and 599 proteins were significantly differentially regulated in murine *Mpo*^*−/−*^ myeloid cells (**B**). GO biological process analysis revealed common enrichment in innate immune response, inflammatory response and response to oxidative stress in both species (**C**, **D**). However, species-specific differences were observed, with human samples showing increased enrichment in humoral immune response, complement acitivtion and coagulation regulation (**C**), whereas murine cells exhibited enrichment in phagocytosis, neutrophil degranulation, and chemotaxis (**D**). Reactome pathway analysis showed limited overlap, with shared enrichment in neutrophil degranulation and innate immune response (**E**, **F**). Human samples were predominantly enriched in MAP kinase, Toll-like receptor and Interleukin signaling pathways (**E**), while murine cells displayed increased Rho GTPase signaling (**F**), highlighting divergent cellular signaling mechanisms between both species. Results are shown for MPO^low^ (*n* = 5) vs control PMNs (*n* = 5) and *Mpo*^*−/−*^ (*n* = 20) vs WT PMNs (*n* = 5).

PMNs from five MPO^low^ patients with the lowest neutrophil MPO activity were selected for proteomic analysis and compared to five randomly selected, non-stratified controls. For the murine experiments, PMNs were isolated from five *Mpo*^−/−^ mice and compared to five littermate controls, ensuring a shared genetic background. (**Sup**. Fig. S5A–D). Purity of isolated PMNs was assessed by flow cytometry (∼95% human, ∼75% murine) **(Sup**. Fig. S6A–C**)**. Comparative proteomic analysis identified 204 differentially expressed proteins out of 2084 detected proteins in human MPO^low^ PMNs and 599 out of 4265 in murine Mpo^−/−^ PMNs (Fig. 3A, 3B, S7). Gene ontology (GO) analysis revealed in both shared upregulation of innate immune responses, inflammatory signaling, and oxidative stress pathways, which underscores conserved aspects of MPO's role in neutrophil biology. However, species-specific signatures were also evident. Human MPO^low^ PMNs exhibited stronger enrichment in humoral immune responses (GO:0006959), complement activation (GO:0006956), coagulation (GO:0050818), and hemostasis-related pathways (GO:1900046), whereas proteoms of murine *Mpo*^−/−^ PMNs were enriched in pathways associated with phagocytosis (GO:0006909), neutrophil degranulation (GO:0043313), and neutrophil chemotaxis (GO:0030593) (Fig. 3C and D).

These differences were also reflected in Reactome pathway analysis. Although both species showed enrichment in neutrophil degranulation and general innate immune system pathways, human MPO^low^ cells were predominantly enriched for multiple MAP kinase and Toll-like receptor (TLR) signaling cascades, including GRB2:SOS/MAPK (R-HSA-354194), p130Cas/MAPK (R-HSA-372708), MAPK2/MAPK activation (R-HSA-5674135), the TLR4 cascade (R-HSA-166016) and Interleukin-1 signaling (R-HSA-9020702) By contrast, murine *Mpo*^−/−^ PMNs showed a bias toward Rho GTPase Effectors (R-MMU-195258) and signaling by Rho GTPases (R-MMU-194315) (Fig. 3E and F). These findings suggest that while inflammatory and oxidative stress responses are conserved across species, regulatory pathway networks are fundamentally divergent.

**Fig. 4:**
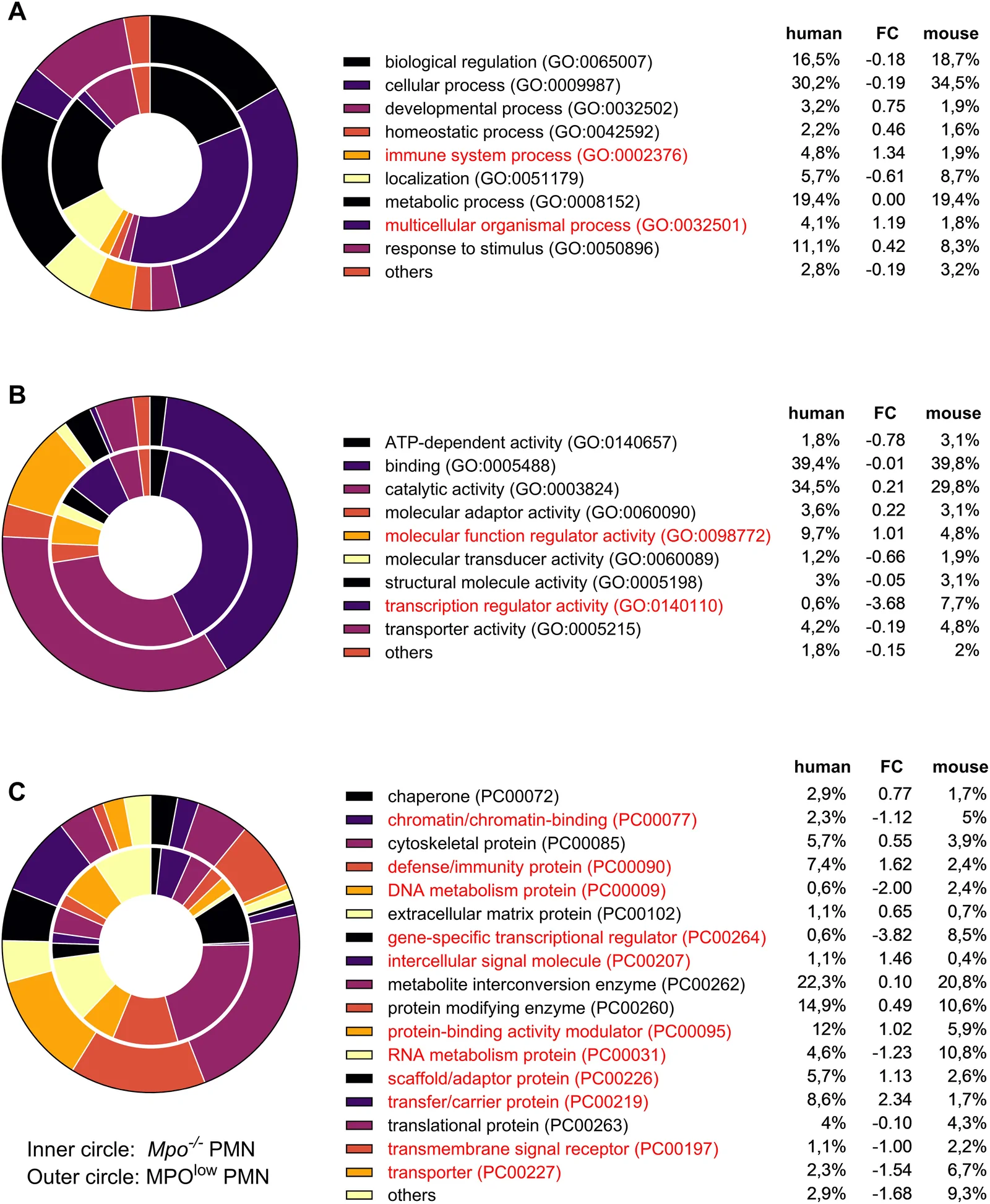
**Comparative proteomics of human MPOlow and murine Mpo^−/−^ neutrophils**. Subclassification of differentially expressed proteins of murine *Mpo*^−/−^ PMN (*n* = 599; inner circle) and human MPOlow PMNs (*n* = 204; outer circle) using PANTHER annotations. Comparative analysis of proteomes revealed no major changes in biological processes (**A**) and molecular function profiles (**B**), but notable differences in protein class profiles (**C**), respectively. PANTHER annotations with a Fold Change [log2] < −1; > 1 are presented in red. Results are shown for MPOlow (*n* = 5) vs control PMNs (*n* = 5) and *Mpo−/−* (*n* = 5) vs WT PMNs (*n* = 5).

PANTHER-subclassification of the murine and human proteomes showed that, at both the molecular-function and biological-process levels, the differentially regulated proteins in the two species mapped to overlapping GO subclasses. Human MPO^low^ cells showed pronounced enrichment in immune system processes (GO:0002376), multicellular organismal processes (GO:0032501), and molecular function regulator activity (GO:0098772), whereas murine *Mpo*^−/−^ PMNs were more enriched for transcription regulator activity (GO:0140110) (Fig. 4A and B). Despite this functional overlap, the underlying protein class distributions diverged significantly between species, suggesting that similar biological functions are mediated by distinct protein sets. Among these were defense/immunity proteins (PC00090), gene-specific transcriptional regulators (PC00264), intercellular signaling molecules (PC00207), scaffold/adaptor proteins (PC00226), transfer/carrier proteins (PC00219), and transmembrane signal receptors (PC00197) (Fig. 4C).

### 3.4 Alterations in MAPK/JNK signaling in MPO-deficient human neutrophils

Several direct and indirect upstream activators of the MAPK/c-Jun N-terminal kinase (JNK) pathway were significantly downregulated in human MPO^low^ PMNs (Fig. 3A–S8). These included adaptor and kinase-associated proteins such as CRKL (Crk-like protein), NCK2 (Non-catalytic region of tyrosine kinase adaptor protein 2), IRAK4 (Interleukin-1 receptor-associated kinase 4), PTEN (phosphatase and tensin homolog), and the upstream kinase MAP2K4 (MKK4). Consistent with these findings, MAP2K4 (MKK4) protein expression, assessed by ELISA, was significantly reduced in MPO^low^ PMNs (0.04% ± 0.01 of total PMN protein) compared to controls (0.09% ± 0.05, p < 0.05) (**Sup**. Fig. S9).

Conversely, RASA2 (RAS p21 protein activator 2), a known RAS inactivator and indirect suppressor of JNK signaling, was significantly upregulated, indicating a suppression of JNK pathway activity. In line with these upstream changes, downstream JNK effectors, including pyroptosis-associated caspases CASP1 and CASP6, were significantly downregulated in MPO^low^ PMNs. An overview of these pathways is shown in **Sup**. Fig. S8. Taken together, these findings highlight a pronounced attenuation of JNK signaling in MPO-deficient human neutrophils, likely affecting stress responses and programmed cell death pathways.

## 4 Discussion

The present study provides a systematic characterization of a cohort of patients with persistently reduced MPO activity and offers epidemiological and mechanistic insights into human neutrophil biology. A total of 11,608 individuals were screened for MPO activity. Of those, 56 patients with reduced MPO activity underwent detailed re-evaluation to distinguish persistently reduced activity from transient hypoactivity. Two distinct subgroups were identified: MPO^low^ (*n* = 12), comprising individuals with persistent MPO deficiency, and MPO^rec^ (*n* = 44), representing those with recovered and only transiently reduced MPO activity. This statistical classification served as the foundation for subsequent molecular analyses.

The prevalence of secondary (i.e., acquired) MPO deficiency was approximately 1:50, whereas primary MPO deficiency occurred at a rate of ca. 1:200. The frequency of primary deficiency in this cohort was consistent with earlier population-based studies from Central Europe reporting a prevalence of approximately 1:400 [25]. Of note, frequencies of MPO deficiency diverge globally, with approximately 1:1000 in Great Britain [26], 1:4000 in Italy [27] and 1:40,000 in Japan [28]. Such variation points to considerable regional heterogeneity, likely driven by genetic, demographic, and methodological influences as well as differences in recruitment strategies and screening approaches.

Whereas earlier studies reported a predominance of primary MPO deficiency, the present work identified a higher proportion of transient, secondary forms [29], which might be attributable to different recruitment strategies. Unlike community-based surveys, the Cologne cohort represents a hospital-based population with a high burden of conditions known to cause transient reductions in MPO activity, including pregnancy, organ transplantation, thromboembolic diseases, hematologic malignancies, severe systemic infections, and iron deficiency [30]. Accordingly, an elevated frequency of secondary deficiency likely reflects enrichment for these clinical states rather than a genuine population-level shift in prevalence.

Prior studies have shown reduced neutrophil MPO protein content in individuals with primary MPO deficiency compared to controls [31], which is consistent with our results. Here, the MPO^low^ group comprised patients with partial MPO deficiency, characterized by markedly reduced but detectable protein levels, and two individuals with complete deficiency, in whom no MPO protein was detectable. A central objective of this study was to examine the relationship between neutrophil MPO expression, enzymatic activity, and circulating MPO plasma concentrations. Persistent MPO deficiency was generally associated with reduced cellular MPO expression, diminished enzymatic activity, and lower plasma MPO levels.

However, in the overall cohort MPO activity correlated only weakly with MPO protein expression, while subgroup analyses revealed no consistent correlation and a broad distribution of MPO activity-to-protein ratios. This heterogeneity suggests that MPO deficiency is mechanistically diverse. In some MPO-deficient individuals, reduced enzymatic activity may primarily reflect low MPO expression, whereas in others, impaired activity may result from qualitative impairments such as defective maturation or dysfunctional enzyme structure rather than protein scarcity alone. This is supported by genetic studies showing that many MPO mutations impair protein maturation, folding, or catalytic activity without reducing transcription or overall protein abundance [32]. Overall, these findings support the concept that MPO deficiency is a heterogeneous disorder encompassing both quantitative and qualitative defects.

Pathway enrichment analyses of MPO^low^ PMNs revealed downregulation of proteins associated with programmed cell death, Toll-like receptor (TLR) cascades, and mitogen-activated protein kinase (MAPK) signaling, with particular emphasis on the JNK axis. Given that JNK acts downstream of TLR4 to promote NADPH oxidase (NOX)-dependent NET formation, and that pharmacological inhibition of either pathway suppresses NETosis [6,33], the altered signaling observed in MPO^low^ PMNs, together with differences in neutrophil degranulation, may affect their pro-inflammatory functions. The potential consequences of these functional alterations would be worthwhile to investigate in future studies.

Several studies have described differences between human and murine PMNs: human neutrophils contain 2- to 10-fold more MPO per cell than murine neutrophils and produce correspondingly higher amounts of HOCl. In addition, the two species differ in granule composition, receptor repertoires, and intracellular signaling landscapes [34]. Our comparative proteomics analyses confirmed this divergence. Human MPO^low^ neutrophils exhibited differential expression of 204 of 2084 proteins, enriched in pathways related to humoral immunity, complement activation, coagulation and MAPK/cytokine signaling. Key changes included downregulation of CRKL, NCK2, IRAK4, PTEN and MKK4 alongside altered regulation of CASP1 (**Sup** Fig. 8 and 9). In contrast, murine Mpo^−/−^ neutrophils displayed 599 of 4265 proteins differentially expressed, enriched in phagocytosis, degranulation, chemotaxis and Rho-GTPase signaling.

The mechanisms underlying MPO's influence on the cellular proteome are diverse and are scarcely investigated. Given that MPO is produced early during granulopoiesis, direct effects on transcription during neutrophil maturation might be conceivable [35,36]. However, MPO-derived oxidants are known to modify and degrade proteins in the mature neutrophil [37], so altered post-translational modifications might be possible. Dedicated research is needed to clearly identify the underlying processes.

The divergent signaling pathways might explain several paradoxical findings between murine and human studies targeting MPO. In LDLR-deficient mice lacking MPO, a ≈50% increase in atherosclerotic lesion size was observed, despite extensive human data identifying MPO as a pro-atherogenic factor. Mechanistically, MPO and HOCl-modified LDL co-localize within human atherosclerotic plaques, in mice HOCl-mediated oxidation in the presence of PMN is barely detectable [15]. Murine lesion progression therefore appears driven more by altered immune-cell behaviour than by oxidative mechanisms illustrating that MPO deficiency may exert fundamentally different pathological consequences across species.

In heart failure, MPO-driven endothelial nitric oxide depletion, extracellular matrix oxidation and remodeling are central pathological mechanisms. Paradoxically, while murine post-MI models typically show benefit from MPO inhibition [16], human trials with MPO inhibitors have yielded inconsistent or modest results [18]. These species-specific discrepancies highlight a critical limitation of preclinical data: Translation from rodents to humans requires careful consideration of divergent MPO-dependent disease mechanisms.

On a more general note, the discrepancy between MPO inhibition and MPO deficiency may also reflect MPO's catalytic versus non-catalytic properties: mechanism-based MPO inhibitors covalently target the heme group and thus abolish catalytic activity [38], whereas the non-enzymatic functions of MPO, including chromatin binding and structural contributions to NET formation, are likely not affected [36,39]. Genetic MPO deficiency, in contrast, may compromise both.

Based on this integrated analysis, primary MPO deficiency stands out as a heterogeneous genetic disorder with higher prevalence in a cohort collected in a tertiary care hospital than previously reported in population-based studies. Affected individuals display a variable reduction in neutrophil MPO expression, enzymatic activity and plasma levels. Proteomic analysis of human MPO^low^ neutrophils implicates suppression of the TLR/JNK axis and downregulation of effectors associated with NETosis and pyroptosis, linking MPO deficiency to impaired neutrophil extracellular trap formation and altered humoral and complement responses.

Comparative murine data indicate that, whereas MPO's oxidant chemistry is largely conserved, species-specific signaling differences may contribute to divergent phenotypes in certain disease models. This information may help to further refine the design of future clinical studies aiming at pharmacologically modulating this central innate pathway of immune activation across the spectrum of vascular inflammatory disease.

### 4.1 Limitations

This study has several limitations. First, the human and murine models are not directly equivalent, as the human MPO^low^ cohort retains residual MPO expression, whereas *Mpo*^*−/−*^ mice represent complete genetic MPO deficiency, limiting direct cross-species comparability. However, concordant vascular and immunomodulatory effects of genetic MPO deficiency and partial pharmacological MPO inhibition in preclinical models suggest that residual MPO activity alone is unlikely to explain the limited clinical translation of MPO-targeted therapies, whereas species-specific differences in neutrophil biology and inflammatory signaling may be more relevant. Second, murine PMN preparations had lower purity than human preparations (∼75% vs. ∼95%), which likely reduced sensitivity in the murine model rather than introducing species-specific differences, as murine and human PMNs were compared within their respective species. Third, cross-species comparisons remain limited because murine and human datasets could only be compared indirectly through annotation-based analyses. Fourth, the TMB-based MPO activity assay is not fully MPO-specific and may detect other peroxidases, potentially overestimating MPO activity, particularly in the MPO^low^ cohort.

## CRediT authorship contribution statement

**Maysam Ahdab:** Conceptualization, Data curation, Formal analysis, Methodology, Investigation, Writing – original draft, Visualization. **Henning Guthoff:** Conceptualization, Formal analysis, Methodology, Project administration, Resources, Writing – original draft. **Nathalie Nix:** Formal analysis, Methodology, Investigation, Visualization. **Sonja Derman:** Conceptualization, Investigation, Project administration, Resources. **Anna Greta Barbe:** Methodology, Resources. **Harshal Nemade:** Investigation, Methodology, Software, Validation. **Elvina S. Philip:** Investigation, Methodology, Software, Writing – review & editing. **Kezia Singgih:** Formal analysis, Investigation, Software, Validation. **Kathrin Nellessen:** Data curation, Investigation, Methodology. **Alina Hüttermann:** Investigation, Methodology, Validation. **Adnana Paunel-Görgülü:** Conceptualization, Project administration, Resources. **Chris Diekmann:** Investigation, Data curation. **Friedrich F. Hoyer:** Resources, Supervision. **Patrick Schelemei:** Formal analysis, Investigation, Methodology, Writing – review & editing. **Jan-Wilm Lackmann:** Conceptualization, Data curation, Investigation, Formal analysis, Resources. **Stefan Müller:** Data curation, Investigation, Methodology, Resources, Software, Visualization, Validation. **Wibke Johannis:** Resources, Investigation, Methodology. **Thomas Streichert:** Resources, Investigation, Methodology. **Stephan Baldus:** Resources, Supervision, Validation, Conceptualization. **Holger Winkels:** Conceptualization, Resources, Supervision, Validation. **Philipp von Stein:** Conceptualization, Formal analysis, Resources, Validation. **Martin Mollenhauer:** Conceptualization, Funding acquisition, Methodology, Validation, Supervision, Resources. **Alexander Hof:** Conceptualization, Project administration, Supervision, Visualization, Writing – original draft, Investigation, Methodology.

## Funding

A. Hof was supported by the German Heart Foundation (F29/21) and the Cologne FORTUNE Programme (248/2021). German Research Foundation Grant No. 397484323 - CRC/TRR259 was given to MM (C03) and HW (A09). M.A. was supported by the Dresdner Herz-Kreislauf-Tage Foundation and the Cologne FORTUNE Programme (72/2024).

## Declaration of competing interest

The authors declare that they have no known competing financial interests or personal relationships that could have appeared to influence the work reported in this paper.
