# Supplementary material for: Comparative analysis of myeloperoxidase deficiency in humans and mice: Conserved and species-specific effects on neutrophil biology
Source: Redox Biol. 2026 Sep 9;97:104386. doi: 10.1016/j.redox.2026.104386 (PMC13595006; doi:10.1016/j.redox.2026.104386)
Supplement: Supplementary file 1 [file mmc1.docx]

**Supplementary Figure 1: Frequency distribution of neutrophil MPO content in the patient and control cohort.** The distribution of neutrophil MPO content (% of total PMN protein) was analyzed in 56 patients with reduced MPXI scores. Measured values displayed a bimodal distribution, indicating the presence of two underlying subpopulations. Accordingly, the data were modeled using a Gaussian mixture model (GMM) consisting of two Gaussian distributions *R²* = 0.8146 (**A**). MPO-competent controls recruited from the outpatient clinic of the University Hospital Cologne (*n* = 20) showed a unimodal distribution of neutrophil MPO content and were approximated using a single Gaussian distribution *R²* = 0.9058 (**B**).

**Supplementary Figure 2: Western blot analysis of MPO expression in PMN.** MPO was clearly detectable in MPO^rec^ and controls whereas in MPO^low^ patients MPO bands were reduced and in two patients completely absent. Equal protein loading was confirmed using GAPDH as a loading control. Measurements with deficient loading control were repeated, as shown in Western blot 7. Results are presented for MPO^rec^ (*n* = 44), MPO^low^ (*n* = 12) and control patients (*n* = 20).

**Supplementary Figure 3: Correlation analyses of MPO content, activity and inflammatory markers.** Photometric analysis by ELISA and densitometric analysis by immunoblotting were conducted to assess MPO protein expression. A correlation analysis between ELISA and immunoblotting revealed a significant positive correlation (**A**). A further correlation analysis between neutrophil MPO content and MPO peroxidase activity also demonstrated a significant positive correlation (**B**). NO-redox potential measurement was performed on isolated PMN to asses MPO-dependent NO consumption. Results are shown for MPO^low^ (*n* = 9), MPO^rec^ (*n* = 27) and controls (*n* = 10) (**C**). Correlation analysis between TMB assay and NO-redox potential measurement showed a significant positive correlation (**D**). Additionally, neutrophil MPO content correlated positively with MPO plasma levels (**E**). MPO-activity/protein ratios did not differ significantly between the study groups (**F**). The number of eosinophilic erythrocytes in the blood count was comparable across all study groups, thereby excluding differential eosinophil-derived peroxidase activity as a potential confounder of the TMB assay (**G**). CRP (**H**) and IL-6 plasma concentrations (**I**) were quantified, revealing no significant differences between the study groups. Results are shown for MPO^rec^ (*n* = 44), MPO^low^ (*n* = 12) and control patients (*n* = 20). **** *p* < 0,0001; *** *p* < 0,001; ** *p* < 0,01; * *p* < 0,05.

**Supplementary Figure 4: Sanger sequencing of MPO exon 10 in an MPO^low^ patient.** Representative Sanger sequencing chromatograms of MPO exon 10 from a wild-type (WT) reference sequence (top) and an MPO^low^ patient (ID 1; bottom). Compared with the WT sequence, the patient carried the c.1705C>T substitution in exon 10, resulting in the missense variant p.Arg569Trp (R569W), which has been associated with hereditary MPO deficiency. The affected amino acid is highlighted in red.

**Supplementary Figure 5: Neutrophil MPO protein expression and plasma levels in MPO^low^ vs control patients and *Mpo^-/-^* vs WT mice used for proteomic analysis.** Neutrophil MPO protein expression and MPO plasma levels were determined by ELISA in human (**A + B**) and in murine samples (**B + C**). Proteomic analysis was performed using PMNs from five MPO^low^ patients selected based on the lowest neutrophil MPO activity within the study cohort and compared with five randomly selected MPO-competent controls. The selected control donors did not significantly differ from the overall control population with respect to neutrophil MPO content or plasma MPO levels. Results are shown for MPO^low^ (*n* = 5) vs control patients (*n* = 5) and *Mpo^-/-^* (*n* = 5) vs WT mice (*n* = 5). **** *p* < 0,0001; *** *p* < 0,001; ** *p* < 0,01; * *p* < 0,05.

**Supplementary Figure 6: Flow cytometric assessment of PMN purity following isolation from mouse bone marrow and human blood.** Representative flow cytometry gating strategy for murine PMNs isolated from bone marrow. Live cells were identified by exclusion of DAPI-positive cells and gated on CD45⁺ leukocytes, followed by identification of PMNs as CD11b⁺Ly6G⁺ cells **(A)**. Representative flow cytometry gating strategy for human PMNs isolated from peripheral blood. Viable leukocytes were identified by exclusion of FVS780-positive cells and gated on CD45⁺ cells, followed by identification of PMNs as CD15⁺CD66b⁺ cells **(B)**. Quantification of PMN purity after isolation, expressed as the percentage of PMNs among live cells. Human PMN preparations consistently achieved approximately 95% purity, whereas murine bone marrow PMN preparations reached approximately 75% purity. Each symbol represents an individual biological sample **(C).**

**Supplementary Figure 7:** **Proteomic profile of MPO^low^ (*n* = 5) and control PMNs (*n* = 5) presented as a heatmap.** Out of 2,084 proteins analyzed, 204 were significantly differentially expressed, defined by Fold Change (FC) [log2] < -0.5; > 0.5 and False Discovery Rate (FDR) < 0.5. Differentially expressed proteins were hierarchically clustered using the Ward method.

 **Supplementary Figure 8: Altered JNK signaling in MPO^low^ PMNs.** Upregulated proteins are shown in orange, downregulated proteins in purple. A variety of stressors including oxidative stress, ultraviolet (UV) radiation, pathogens, inflammatory cytokines, and growth factors can activate the JNK signaling pathway. Signal transduction occurs via G-protein-coupled receptors (GPCRs), receptor tyrosine kinases (RTKs), Toll-like receptors (TLRs) and 4cytokine receptors, which activate MAP3-kinases through associated carrier proteins and GTPases. A subsequent phosphorylation cascade proceeds via MAP2-kinases, culminating in activation of the effector kinase JNK. JNK phosphorylates various transcription factors and cytoplasmic signaling proteins, thereby modulating processes such as cell death, cell proliferation, cell differentiation, and inflammatory responses. Both direct and indirect activators of the JNK pathway were downregulated in MPO^low^ (*n* = 5) compared to controls (*n* = 5), whereas indirect inhibitors, such as RASA2 (RAS p21 Protein Activator 2), were upregulated. In addition, effector caspases of pyroptosis (caspase 1 and 6) and PTEN (Phosphatase and Tensin Homolog), a key inhibitor of the PI3K–Akt signaling pathway, were underexpressed in MPO^low^ PMNs.

**Supplementary Figure 9: Neutrophil MKK4 protein expression in MPO^low^ and control patients.** MKK4-ELISA was conducted on isolated PMN to determine MKK4 protein expression. Results are shown for MPO^low^ (*n* = 5) and control patients (*n* = 5). **** *p* < 0,0001; *** *p* < 0,001; ** *p* < 0,01; * *p* < 0,05.

**Supplementary Table 1. PCR and Sanger sequencing primers for MPO gene analysis.** Primer sequences, target regions, annealing temperatures, and PCR product sizes used for amplification and sequencing of the MPO gene are shown. Three overlapping PCR fragments covering exons 1-6, 8-10, and 11-12 were generated, followed by Sanger sequencing using the indicated sequencing primers.

**Supplementary Table 2: General overview of mechanisms and clinical features of MPO deficiency.** MPO deficiency in PMNs and monocytes may result from either reduced enzyme expression or impaired enzymatic activity, leading to diminished production of reactive oxidants such as hypochlorous acid (HOCl) from hydrogen peroxide (H₂O₂). Primary, chronic MPO deficiency is caused by inherited germline point mutations (e.g., R569W/Y173C, M251T, G501S, R499C or a 14-base deletion in exon 9). Secondary, transient MPO deficiency arises from acquired conditions affecting myelopoiesis or enzyme function, including infections, metabolic disorders, pregnancy, hematologic malignancies, drug exposures and toxic insults. Clinically, most individuals (>95%) remain asymptomatic. However, a subset may develop mild, non-specific infections, mucocutaneous or invasive *Candida* infections, recurrent bacterial infections, pustular dermatoses, chronic inflammatory or rheumatologic-like symptoms. Notably, MPO deficiency has also been associated with a reduced atherosclerotic plaque burden.^37-42^

Additional References:

[41]	F. Lanza, Clinical manifestation of myeloperoxidase deficiency, J. Mol. Med. (Berl). 76(1998)676–681.

[42] C.Nguyen, H.P.Katner, Myeloperoxidase deficiency manifesting as pustular candidal dermatitis, Clin. Infect. Dis. 24(1997)258–260.

[43] M. F. Parry, et al., Myeloperoxidase deficiency: prevalence and clinical significance, Ann. Intern. Med. 95(1981)293–301.

[44] S. Mousavi, M. H. Hosseini, S. Sarandili, B. Nejati, Myeloperoxidase deficiency: a rare

case, Cureus 16(2024).

[45] M. Vergnano et al., Loss-of-Function myeloperoxidase mutations are associated with increased neutrophil counts and pustular skin disease, Am. J. Hum. Genet.107(2020)539.

**Supplementary Table 3: Clinical records of two MPO^low^ individuals with no detectable MPO protein.** In both cases, MPO protein levels were below the detection threshold of the quantitative MPO ELISA (<0.08% of total PMN protein). Consistently, MPO immunoblot analysis demonstrated complete absence of MPO-specific bands. Neither individual showed a clinical history suggestive of severe innate immunodeficiency, recurrent opportunistic infections, or invasive fungal disease. Routine hematologic parameters were largely unremarkable in both individuals.
